# Visual Exploration at Higher Fixation Frequency Increases Subsequent Memory Recall

**DOI:** 10.1093/texcom/tgaa032

**Published:** 2020-07-21

**Authors:** Bernhard Fehlmann, David Coynel, Nathalie Schicktanz, Annette Milnik, Leo Gschwind, Pascal Hofmann, Andreas Papassotiropoulos, Dominique J-F de Quervain

**Affiliations:** 1 Division of Cognitive Neuroscience, Department of Psychology, University of Basel, Basel 4055, Switzerland; 2 Transfaculty Research Platform, University of Basel, Basel 4055, Switzerland; 3 Division of Molecular Neuroscience, Department of Psychology, University of Basel, Basel 4055, Switzerland; 4 Department Biozentrum, Life Sciences Training Facility, University of Basel, Basel 4056, Switzerland; 5 University Psychiatric Clinics, University of Basel, Basel 4002, Switzerland

**Keywords:** encoding, eye fixations, fMRI, medial temporal lobe, memory

## Abstract

Only a small proportion of what we see can later be recalled. Up to date it is unknown how far differences in visual exploration during encoding affect the strength of episodic memories. Here, we identified individual gaze characteristics by analyzing eye tracking data in a picture encoding task performed by 967 healthy subjects during fMRI. We found a positive correlation between fixation frequency during visual exploration and subsequent free recall performance. Brain imaging results showed a positive correlation of fixation frequency with activations in regions related to vision and memory, including the medial temporal lobe. To investigate if higher fixation frequency is causally linked to better memory, we experimentally manipulated visual exploration patterns in an independent population of 64 subjects. Doubling the number of fixations within a given exploration time increased subsequent free recall performance by 19%. Our findings provide evidence for a causal relationship between fixation frequency and episodic memory for visual information.

## Introduction

Visual episodic memory consists of the voluntary recollection of previously encoded visual information along with contextual information. As such, visual episodic memory fundamentally depends on the visual sampling during encoding. Visual sampling is temporally restricted to phases of steady gaze referred to as fixations (Ross et al. 2001), and fine-grained information is spatially bound to the visual field projected to the fovea (Henderson 2003). As a consequence, only a minor fraction of the visual world sampled by an individual builds the basis for memory formation. It is thus crucial to consider visual exploration characteristics as an integrative part of memory processing and to ask how they affect memory encoding and later performance (Voss et al. 2017). Previous studies reported positive interindividual correlations between the number of fixations and memory of objects (Pertzov et al. 2009; Kafkas and Montaldi 2011) and faces (Heisz et al. 2013; Olsen et al. 2016). It is unknown, however, if such a correlation also exists for memory of complex scenes and, importantly, if there is an underlying causal relationship.

In a first experiment, we explored how individual visual exploration characteristics, quantified by the number of fixations, blink duration, and interfixation distance, are related to free recall memory performance across complex scenes. Based on previous findings (Pertzov et al. 2009; Kafkas and Montaldi 2011; Heisz et al. 2013; Olsen et al. 2016), we defined the number of fixations as the variable of primary interest. We analyzed eye tracking data of 967 subjects completing a memory paradigm while controlling for inherent differences in stimulus properties. This enabled us to focus on interindividual exploration differences that are not attributable to the stimulus itself (e.g., saliency (Itti and Koch 2001), complexity (Voss et al. 2017), emotional valence (Sharot et al. 2008), semantic density (Henderson and Hayes 2017), and memorability (Bylinskii et al. 2015)).

Using an fMRI paradigm allowed us to study the relationship between fixation frequency and memory on a neural level. In the context of face recognition, previous studies have linked visual sampling to activity in the medial temporal lobe (MTL; Liu et al. 2017) and to increased memory performance (Olsen et al. 2016). The aim of the first experiment was to combine these findings and to extend them from face recognition to episodic memory and a broad range of complex scenes. In a second experiment, we—for the first time to our knowledge—investigated causality of the found relationship between fixation frequency and memory by manipulating the scan paths of 64 additional subjects during memory encoding.

## Materials and Methods

### Experiment 1

The first experiment was based on a large-scale, simultaneous fMRI and eye tracking study conducted at the University Hospital of Basel, Switzerland (see Heck et al. 2014).

#### Participants

We analyzed data of 1485 subjects (917 females, mean age = 22.34, SD = 3.25, range 18–35) which completed the study. Participants were free of any neurological or psychiatric conditions and did not take any medication at the time of testing (except hormonal contraceptives). Procedures were approved by the ethics committee of the Cantons of Basel-Stadt and Basel-Landschaft.

#### Experimental Procedure

**Figure 1:**
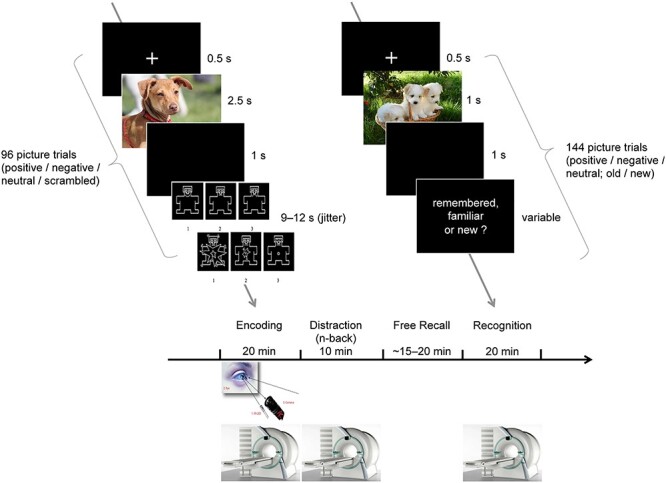
Paradigm of Experiment 1. For 20 min, participants encoded 96 pictures in the fMRI scanner under free viewing conditions while their eyes were monitored by an eye tracker. The pictures were rated in terms of (a) valence and (b) arousal. Scrambled pictures were rated in terms of form (vertical, symmetric, horizontal) and size (small, medium, large) of the geometrical object instead. After 10 min of an *n*-back working memory task that served as a distraction, they were confronted with a free recall memory test of the pictures outside the scanner and without time limit (approx. duration, 15–20 min). Participants were then repositioned in the scanner and performed a recognition task for 20 min. Because of copyright restraints from the IAPS, the pictures in the figure are not the original IAPS pictures used in the study but illustrative pictures that resemble them.

After participants received the general study information and provided written informed consent upon arrival, they were instructed and trained on a picture encoding task. Following training completion, participants were positioned in the scanner for the actual task, lasting for 20 min. Immediately afterwards, they performed an *n*-back working memory task for 10 additional minutes. Having left the scanner, participants were confronted with a surprise free recall memory test of the pictures without time limit. Participants were then repositioned in the scanner and performed a recognition task for 20 min, before structural MRI (T1) and diffusion MRI data were acquired for the remaining 20 min (see Fig. 1). The total length of the experimental procedure ranged from 3 to 4.5 h per subject. Participants were rewarded with 25 CHF/h.

#### Stimuli

Seventy-two color images, divided into three valence groups, served as the main stimuli for the encoding task. On the basis of normative valence scores, 64 pictures from the International Affective Picture System (IAPS; Lang et al. 2008) were assigned to emotionally negative (2.3 ± 0.6), neutral (5.0 ± 0.3), and positive (7.6 ± 0.4) groups. In order to equate the picture set for visual complexity and content (e.g., human presence), 8 neutral pictures were selected from an in-house standardized picture set, resulting in a total of 24 pictures per valence category. Examples of pictures are as follows: erotica, sports, and appealing animals for the positive valence; bodily injury, snakes, and attack scenes for the negative valence; and neutral faces, household objects, and buildings for the neutral condition. Furthermore, 24 scrambled pictures were included. Their background contained the color information of the previously described stimuli, overlaid with a crystal and distortion filter (Adobe Photoshop CS3). In the foreground was one geometrical object, varying between pictures in terms of form, size, position, and orientation. To control for primacy and recency effects in memory, two additional pictures of neutral objects were shown at the beginning and the end of the task respectively, but were discarded from further analyses.

#### Encoding Task

The task was to visually explore 96 pictures under a free viewing condition, that is, without any restrictions. Each of the 96 encoding trials started with a fixation cross, presented for 500 ms against a dark background, and was followed by the presentation of one picture for 2.5 s. The picture onset time was jittered within 3 s (1 repetition time [TR]) per valence category with regard to the scan onset. A blank, dark screen followed the offset of the picture for 1 s. Trials were separated by a variable period of 9–12 s (jitter). During this period, participants rated the emotional valence (negative, neutral, positive) and perceived arousal (low, middle, high) of the meaningful pictures on two separate three-point Likert scales by button press. The scrambled pictures were rated in terms of form (vertical, symmetric, horizontal) and size (small, medium, large) of the geometrical object (see Fig. 1). Across all trials, pictures were presented in a quasi-randomized order, allowing for a maximum of four consecutive pictures with identical valence categories.

#### Free Recall Task

To document their free recall, subjects had to write a short description of each remembered picture. A picture was judged as correctly recalled if two trained investigators independently allocated the description to the same picture from the encoding set (interrater reliability >98%, reflecting the accordance rate between the two investigators across all trials). A third, blinded rater made a final decision for pictures that were rated differently. Free recall performance was assessed by the total number of correctly recalled pictures. We additionally assessed recognition performance but focused on free recall rather than recognition, because for the latter we used subjective remember-know judgments (see Supplementary Material), a procedure that has been questioned as being suited to differentiate between episodic and semantic memory (Wixted 2009; McCabe et al. 2011).

#### Eye Tracking Data Acquisition

During fMRI sessions, the eye movements were recorded using an infrared camera integrated into the goggle system (NordicNeuroLab, Bergen, Norway). The left eye position was sampled at 60 Hz and a spatial accuracy of about 1° (according to the manufacturer). The acquisition was controlled by ViewPoint EyeTracker software (Arrington Research), and calibration was performed following the built-in 9-point procedure at the beginning of the experiments. In total, 967 subjects (596 females, mean age = 22.28, SD = 3.28, range 18–35) had eye tracking data before preprocessing.

#### Eye Tracking Data Preprocessing

Collected raw data were preprocessed in R (v3.3.3; RRID:SCR_ 001905; R Core Team, 2015; http://www.r-project.org/). For each subject, fixation detection was done with an individual, velocity-based algorithm (“saccades” package, Malsburg 2015). Fixations with duration of less than 100 ms and saccades were discarded for further analyses. Slow, drift-like displacements of the recorded fixation coordinates were corrected as follows. The value of correction was calculated for each time point as its displacement relative to a baseline, represented by a moving median with a window size of 3301 sampling points (~55 s). This procedure is roughly familiar to high-pass filtering at 0.008 Hz, but more appropriate for time domain-encoded signals (Smith 2003).

If not indicated otherwise, outlier detection of eye tracking data was based on boxplots. The first (*q*_1_) and third (*q*_3_) quartiles were estimated based on ideal fourths. After determining the interquartile range (*IQR*), a data point x was defined as an outlier if x < *q*_1_–1.5(*IQR*) or *x* > *q*_3_ + 1.5(*IQR*) (Wilcox 2012). Subjects were excluded if they were identified as outliers for calibration data (total gaze deviation from expected grid, *n* = 80) or the eye movement velocity distribution (with respect to the *x*- and/or the *y*-axis, *n* = 87). Additionally, trials with the following characteristics were discarded: only one trial fixation (assuming that at least one saccade had to be made to ensure picture encoding), high signal loss (pupil aspect ratio < 0.5 in over 50% of the picture data samples), and/or pupil profile distortions (low correlation of the pupil response with the grand average profile, see Supplementary Materials and Methods Experiment 1). After removing data of nine additional subjects with no valid trials, further analyses related to eye tracking were based on a total of 791 subjects (475 females, mean age = 22.35, SD = 3.39, range 18–35).

#### Eye Tracking Parameters

All eye movement measures were extracted per subject and picture trial. To quantify visual sampling intensity, the number of fixations at encoding was counted within the 2.5 s of picture presentation (*N*_fix_) and restricted to areas of interest (*N*_fix_ in AOIs). For the definition of different AOIs, fixations of 200 random subjects were sampled per picture. Afterwards, a data-driven method to identify semantic areas of interest was applied (mean shift clustering, “MeanShift” package, Ciollaro and Wang 2016; see Supplementary Materials and Methods Experiment 1). Visual sampling quality was specified by the number of unique AOIs covered by fixations (N_AOIs_). This measure is perfectly inversely correlated with skipped AOIs and represents the completeness by which the distinct semantic aspects of a visual stimulus have been encoded (Holmqvist et al. 2011). However, due to its high redundancy to the number of fixations in AOIs (*N*_fix_ in AOIs; *r* = 0.91; see Supplementary Table 2), the AOIs visited were not further investigated. As measures of secondary interest, visual sampling continuity was measured by the total time the eyes were closed during a complete picture trial (blink duration). Blink detection was based on the geometry of an ellipse fitted to the pupil, a default aspect ratio threshold of 0.6 (ViewPoint, Arrington Research), and a minimum duration of 83 ms (see Van Orden et al. 2000). Visual sampling dispersion was quantified by the average distance between two sequential fixation points across all picture fixations (interfixation distance), elsewhere described as an inverse index of attentional narrowing (Sharot et al. 2008). Importantly, to account for inherent differences in stimulus properties and their effect on visual exploration, all eye tracking parameters were *z*-standardized within pictures.

#### (f)MRI Data Acquisition

MRI imaging was performed using a 3 T Siemens Magnetom Verio whole-body MR unit with a 12-channel head coil. Head movements were minimized by using small cushions and the instruction to lie as still as possible. Blood oxygen level-dependent fMRI was obtained by a single-shot echo-planar sequence using parallel imaging (GRAPPA) with the following parameters: TE (echo time) = 35 ms, FOV (field of view) = 22 cm, acquisition matrix = 80 × 80, interpolated to 128 × 128, voxel size = 2.75 × 2.75 × 4 mm^3^, and GRAPPA acceleration factor *r* = 2.0. With a midsaggital scout image and an ascending interleaved sequence, we acquired 32 contiguous axial slices, placed along the anterior–posterior commissure plane and covering the entire brain with a TR = 3000 ms (α = 82°). The first two acquisitions were discarded due to T1 saturation effects. A high-resolution T1-weighted anatomical image was obtained by a magnetization-prepared gradient echo sequence (MPRAGE) with the following parameters: TR = 2000 ms, TE = 3.37 ms, TI = 1000 ms, flip angle = 8°, 176 slices, FOV = 256 mm, and voxel size = 1 × 1 × 1 mm^3^ (see Heck et al. 2014). Stimuli were presented with Neurobs Presentation (Neurobehavioral Systems, Inc., http://www.neurobs.com) and presented via an MR-compatible goggle system (VisualSystem; NordicNeuroLab, Bergen, Norway). The system provided 800 × 600-pixel resolution with a field of view that nominally spans 23.5° in the vertical direction and 30° in the horizontal direction. Dioptric correction lenses were used when necessary. Responses were collected with an MR-compatible response box.

#### Construction of a Population-Average Anatomical Probabilistic Atlas

The first 1000 participants that participated in the study and passed the T1 quality check were selected as a representative sample of our young and healthy population. Their T1-weighted images were used for the construction of a population-specific probabilistic anatomical atlas. The atlas consists of 35 cortical regions per hemisphere, as well as 17 subcortical regions (for details, see Supplementary Materials and Methods Experiment 1).

#### Preprocessing of (f)MRI Data

If not indicated otherwise, brain imaging data were processed in SPM12 (Statistical Parametric Mapping; v6685; Wellcome Trust Centre for Neuroimaginghttp://www.fil.ion.ucl.ac.uk/spm/) implemented in MATLAB R2016a (MathWorks). Volumes were slice-time corrected to the first slice, realigned using the “register to mean” option, and coregistered to the anatomical image by applying a normalized mutual information 3D rigid body transformation. Successful coregistration was visually verified for each subject. Subject-to-template normalization was done using DARTEL (Ashburner 2007), which allows registration to both cortical and subcortical regions and has been shown to perform well in volume-based alignment (Klein et al. 2009). Normalization incorporated the following four steps: 1) The structural image of each subject was segmented using the “Segment” procedure. 2) The resulting gray and white matter images were used to compute a subject-to-template transformation. The template employed here comes from a subgroup of 1000 subjects, part of which were included in the present experiment (Heck et al. 2014). 3) An affine transformation was applied to map the group template-to-MNI space. 4) Subject-to-template and template-to-MNI transformations were combined to map the functional images to MNI space. The functional images were smoothed with an isotropic 8 mm full-width at half-maximum (FWHM) Gaussian filter. Normalized functional images were masked using information from their respective T1 anatomical file as follows: At first, the three-tissue classification probability maps of the “Segment” procedure (gray matter, white matter, and csf) were summed to define the mask. The mask was binarized, dilated, and eroded with a 3 × 3 × 3 voxels kernel using fslmaths (FSL; v5.0.9; RRID, SCR_002823, Jenkinson et al. 2012) to fill in potential small holes in the mask. The previously computed DARTEL flowfield was used to normalize the brain mask to MNI space, at the spatial resolution of the functional images. The resulting nonbinary mask was thresholded at 50% and applied to the normalized functional images. Consequently, the implicit intensity-based masking threshold usually employed to compute a brain mask from the functional data during the first-level specification (spm_get_defaults(“mask.thresh”) fixed at 0.8) was not needed any longer and set to a lower value of 0.05.

#### Fixations and Memory

After preprocessing the eye tracking data, a subject-specific average value was computed for each eye tracking parameter. This was done separately for each of the three valence categories. If for a given subject and valence category, an average parameter value was based on less than 25% (=6 out of 24 pictures) of the available trials, it was not further considered. For 709 subjects, we had complete memory performance data in addition to the eye tracking data (433 females, mean age = 22.47, SD = 3.46, range 18–35). Data of these subjects entered the analyses, done in R. We applied linear mixed models (“nlme” package, Pinheiro et al. 2019) in combination with ANOVA (SS II). The participant ID was included as the random effect in the mixed models. The dependent variable was the free recall performance. Independent variables were the specified eye movement parameters. Each independent variable was assessed in a separate model, together with the factor picture valence. Sex, age, as well as the factors “goggles” (accounting for a software update of the eye tracker) and “recall room” (accounting for three changes of the room in which the free recall task took place during the course of the experiment) were included as covariates. We tested for main effects of eye movement measures and their interactions with picture valence. In a first step, we assessed significance of the interaction terms of the four full models using an FDR correction over respective *P* values. In case of significant interactions, the main effect is still reported, but while accounting for the interaction term (SS III). In addition, post hoc tests were applied to further investigate the interaction effect. In case of nonsignificant interactions, the model was recalculated without the interaction term. The reported *P* values of the main effects in the final models are again FDR-corrected for multiple comparisons (=4, corresponding to *N*_fix_, *N*_fix_ in AOIs, blink duration, and interfixation distance). *P* values of post hoc tests are FDR-corrected by the number of conducted post hoc tests (=3, corresponding to the three valence categories) within each main model. Effect sizes for repeated measures are indicated by generalized semipartial *R*^2^ (*R*^2^β^*^; Jaeger et al. 2017), a generalization of the widely used marginal *R*^2^-statistics (Nakagawa and Schielzeth 2013) which is comparable to effect size measures of between-subject designs. *R*^2^β^*^ > 0.01, *R*^2^β^*^ > 0.09, and *R*^2^β^*^ > 0.25 are considered small, intermediate, and large effects, respectively.

#### Fixations and fMRI: First-Level Analyses

To investigate the relation between the number of fixations at encoding and functional brain activity, we conducted a parametric modulation analysis. Thereby, we only considered subjects with data for both fMRI and number of fixations (*N* = 775; 463 females, mean age = 22.37, SD = 3.40, range 18–35) and defined a general linear model (GLM) on the individual level. The following regressors were included: picture presentation, rating scale presentation (separately for all three valence categories and scrambled pictures), and button presses. Picture and rating scale presentation were modeled by a boxcar function of constant duration, whereas button presses were modeled by a delta function at press onset. Mean-centered number of fixations per trial was included as a linear parametric modulator, for each picture category separately. The regressors were convolved with the canonical hemodynamic response function (HRF). Intrinsic autocorrelations were accounted for by AR(1), and low-frequency drifts were removed via high-pass filtering (time constant 128 s). The final design matrix was completed with six movement parameters obtained from spatial realignment. We aimed at identifying brain activity related to numbers of fixations, independently of valence. Therefore, we contrasted the parametric regressors of the three emotional valences versus the scrambled condition (referred to as parametric all vs. scrambled contrast), which served as a baseline [+1, +1, +1, −3].

#### Fixations and fMRI: Second-Level Analyses

The subject-specific contrast estimates from the first-level analyses entered the second-level group analyses as dependent variables. Sex, age, as well as the factor “goggles” (accounting for a software update of the eye tracker) were included as covariates into the GLM. The main effect of the parametric modulator “number of fixations” was assessed with a linear model. The statistical threshold of the two-sided hypothesis was set using family-wise error (FWE) correction for multiple comparisons across the whole brain (WB) at the voxel level at *p*_FWE-WB_ < 0.05 (corresponding to *t*(771) ≥/≤ ±4.82).

#### fMRI and Memory: First-Level Analyses

We additionally analyzed whether brain activation in clusters associated with the number of fixations at encoding was related to memory performance. This was done on the basis of 1395 subjects with valid data for fMRI and the free recall task (854 females, mean age = 22.40, SD = 3.27, range 18–35). Unlike the parametric model, the underlying model does not contain any eye tracking regressors as parametric modulators but is otherwise identical (the model contains the following regressors: picture presentation, rating scale presentation, and button presses, convolved with the HRF, as well as six movement parameters). On the first level, the difference between the parameter estimates of the three emotional valences and the scrambled condition (referred to as all vs. scrambled contrast) were calculated for each subject and voxel [+1, +1, +1, −3].

#### fMRI and Memory: Second-Level Analyses

The subject-specific contrast estimates from the first-level analyses entered the second-level group analyses as dependent variables. Sex, age, as well as the factors “gradient” (accounting for two changes of gradient coils), “software” (accounting for a change in scanner software), and “recall room” (accounting for three changes of the room in which the free recall task took place during the course to the experiment) were included as covariates. The free recall performance was entered as the independent variable of interest, and its association with brain activity was assessed with a linear model. Because we were only interested in voxels previously showing an association between the encoding signal and the number of fixations in AOIs, the statistical threshold of the two-sided hypothesis for the FWE correction at voxel level was adjusted accordingly (*p*_FWE-SVC_ < 0.05, corresponding to *t*(1388) ≥/≤ ±4.03).

### Experiment 2

The second experiment was based on an eye tracking study conducted at the University of Basel, Switzerland.

#### Participants

We collected data of 66 subjects (33 females; mean age = 23.30, SD = 3.89, range 18–32) which did not participate in Experiment 1, were free of any neurological or psychiatric conditions, and did not take any medication at the time of testing (except hormonal contraceptives). The experiment was approved by the ethics committee of the Cantons of Basel-Stadt and Basel-Landschaft.

#### Experimental Procedure

**Figure 2:**
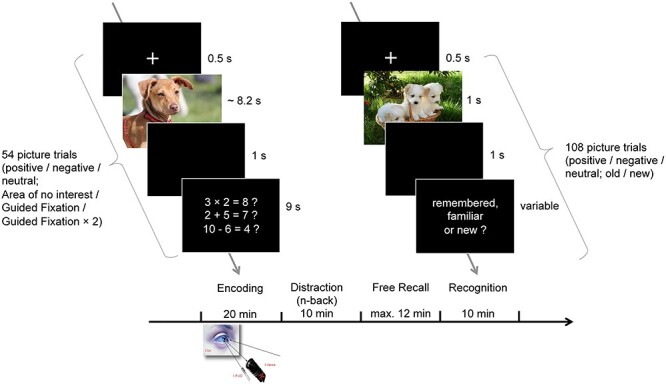
Paradigm of Experiment 2. For 20 min, participants encoded 54 pictures under guided viewing conditions while their eyes were monitored by an eye tracker. Thereby, a quasi-random subset of 18 pictures had to be encoded under “Area of no Interest,” the “Guided Fixation,” and the “Guided Fixation × 2” conditions, respectively. After 10 min of an n-back working memory task that served as a distraction, they were confronted with a free recall memory test of the pictures with an upper time limit of 12 min, followed by a recognition task of 10 min. Because of copyright restraints from the IAPS, the pictures in the figure are not the original IAPS pictures used in the study but illustrative pictures that resemble them.

Participants received the general study information and gave their written informed consent upon arrival. They were then briefed and trained on a guided picture encoding task. The completion of the encoding task per se took 20 min and was followed by 10 min of an n-back working memory task that served as a distraction. Immediately afterwards, participants were confronted with a free recall memory test of the pictures with an upper time limit of 12 min. Finally, participants performed a recognition task for 10 min (see Fig. 2). The experiment took a total time between 1.25 and 2 h per subject and was rewarded with 25 CHF/h.

#### Stimuli

From the 72 photographs of Experiment 1, a subset of 54 pictures was used, including their predefined AOIs (see Supplementary Materials and Methods Experiment 1). All the pictures from the original set with a maximum of two AOIs were thereby discarded. To arrive at an equal amount of 18 pictures per valence category, 1 negative picture with a high number of AOIs (=9) and 4 negative pictures with the most redundant content were additionally excluded. Each of the 54 chosen pictures had a specific number of AOIs (AOIs_pic_), ranging from 3 to 8. The pictures were divided into 3 sets, each containing 18 pictures matched in terms of valence category (6 negative, neutral, and positive pictures, respectively), number of AOIs, and free recall memorability (i.e., the likelihood that a picture was recalled across participants) based on the data of Experiment 1. The same four pictures as in Experiment 1 served to control for primacy and recency effects and were not further analyzed.

#### Encoding Task

The task was to visually explore 54 pictures under guided viewing conditions. Each of the 54 encoding trials started with a fixation cross presented for 500 ms against dark background and was followed by the presentation of one picture for an average of 8.2 s. Afterwards, the participants had to evaluate simple arithmetic operations that could either be correct (33%; e.g., 2 + 5 = 7) or wrong (67%; e.g., 3 × 2 = 8) for 9 s (see Fig. 2). This distractor task was chosen instead of the picture ratings in Experiment 1. It was assumed that rating tasks related to the content of the picture could provoke scanning patterns deviating from the guided viewing in Experiment 2 and would therefore be less suitable than in Experiment 1. Across all trials, pictures were presented in a quasi-randomized order, allowing for a maximum of four consecutive pictures with identical valence categories and/or being of the identical set.

#### Guided Viewing Conditions

**Figure 3:**
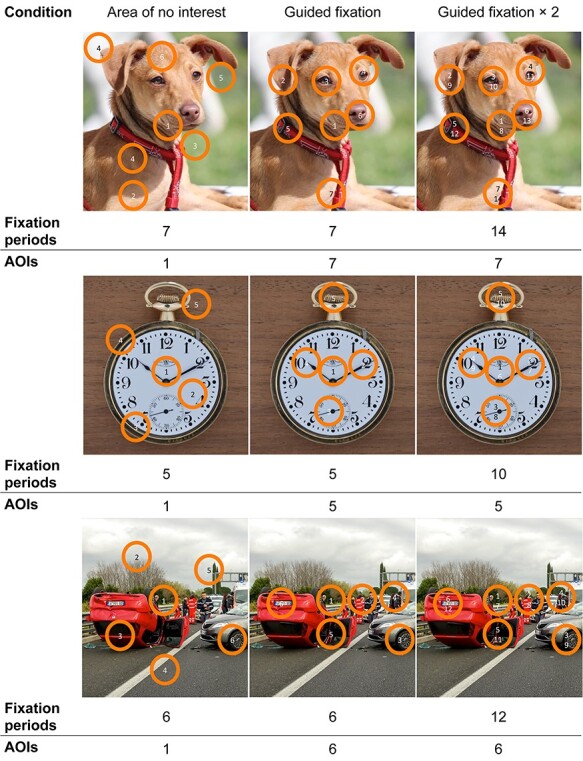
Experimental manipulation of fixation frequency and location. The task was to follow the path of a moving circle (1-2-3-...) within 54 presented pictures. Paths varied between conditions. Top left: circle covering 1 AOI (1) and 6 areas of no interest (2–7), resulting in 7 fixation periods. Top center: 7 AOIs, each covered by the circle once, resulting in 7 fixations periods. Top right: 7 AOIs, each covered by the circle twice, resulting in 14 fixation periods. Scanning time was constant across conditions. AOI 1 was related to the starting position in the middle of the picture. AOIs and areas of no interest were derived from the empirical data of Experiment 1 (see Supplementary Materials and Methods Experiment 2). Middle and lower rows: further examples of neutral (“watch”) and negative (“car accident”) pictures, including their AOIs and areas of no interest. Because of copyright restraints from the IAPS, the pictures in the figure are not the original IAPS pictures used in the study but illustrative pictures that resemble them.

Importantly, for each participant, the three picture sets were randomly assigned to three guided viewing conditions, referred to as “Area of no Interest,” “Guided Fixation,” and “Guided Fixation × 2” (see Fig. 3). The instruction for all conditions was to follow the path of a moving circle and focus exclusively on the content lying within while ignoring the rest of the picture. The circle alternated between phases of steady state similar to fixations periods and fast movements imitating saccade-like movements. In the “Area of no Interest” condition, the pathway of the circle included *n* = AOIs_pic_ fixation periods, but initially only covered one AOI in the center of the picture, while the subsequent fixation periods (AOIs_pic_ − 1) were lying outside all AOIs. In the “Guided Fixation” condition, the circle stopped at each AOI exactly once, corresponding to *n* = AOIs_pic_ fixation periods. Finally, in the “Guided Fixation × 2” condition, the pathway of the circle had *n* = AOIs_pic_ × 2 fixation periods, allowing it to cover each AOI exactly twice.

The centroids of the AOIs were derived from Experiment 1. Centroids of areas of no interest were calculated in a similar way (see Supplementary Materials and Methods Experiment 1), but based only on fixations that were not in AOIs. The size of the moving circle represents the average size of an AOI in Experiment 1. The moving characteristics of the circle were also based on the data of Experiment 1, with the intent of roughly mimicking plausible eye movement patterns. The velocity of the circle therefore alternates between 0°/s, imitating fixations, and 200°/s, imitating saccades. The latter is corresponding to an estimation of the peak saccadic velocity for the maximal possible angular distance of 30° (Boghen et al. 1974) between two AOIs in the current experiment. Depending on the number of AOIs (AOIs_pic_) and the doubled fixation periods in the “Guided Fixation × 2” condition, the amount of fixation periods per picture ranges from 3 to 16. The duration of these fixation periods is inversely related to their number, varying between 2333.33 ms for AOIs_pic_ = 3 and 437.50 ms for AOIs_pic_ = 16. Based on the distribution of the expected fixation frequencies (see Supplementary Fig. 1), the average fixation period (i.e., the duration the circle stayed at one point before moving to the next) was 1608.52 ms (SD = 475.54 ms) in the “Area of no interest” condition, 1606.61 ms (SD = 476.43 ms) in the “Guided Fixation” condition, and 736.04 ms (SD = 238.50 ms) in the “Guided Fixation × 2” condition. The lower threshold was set based on the median fixation duration of 436.04 ms in Experiment 1 and ensured that the encoding of any given AOI was still easily possible. This setting was chosen in order to allow all pictures to be fixated and thus encoded for the same amount of time (7000 ms) and thereby to avoid mere effects of exposure time. Depending on the different lengths of saccade paths, the actual picture presentation varied between 7712 and 9312 ms (*M =* 8153.63 ms, SD = 276.77 ms). The order of the AOIs was quasi-randomized for each subject and picture. The first restriction is with regard to the first fixation period always starting at the center of the picture, where the fixation cross had been previously located. The second restriction is with regard to the “Guided Fixation × 2” condition, where the scan path first covered each AOI first once in random order and then recapitulated itself to cover each AOI a second time. This procedure was chosen to keep the cognitive load and thus the potential risk of interference with the actual memory processes as low as possible, even at minimum fixation durations/maximum fixation periods as present in the “Guided Fixation × 2” condition. The compliance of the subjects and the accuracy with which their gaze could be guided was estimated by correlating the scan path of the moving circle and the actual fixation pattern for each trial separately. In addition, it was characterized by the time lag between the moving circle arriving at a new picture region and the first fixation within this region. The fixations were thereby searched in a time window of 416.67 ms (50 sampling points) after the circle came to a halt.

#### Working Memory and Free Recall Task

The working memory and the free recall task are almost identical to Experiment 1. The free recall had a slightly different timing due to the reduced picture set (see Experimental procedure). The interrater reliability in the free recall task was equally high (>95%). We additionally assessed recognition performance (see Supplementary Material). However, following the reasoning of Experiment 1, we focused on free recall rather than recognition.

#### Eye Tracking Data Acquisition

To investigate how good subjects were able to comply with the three viewing conditions, their eye movements were recorded with an SMI RED device. The gaze position accuracy of this system was 0.4°, the spatial resolution 0.03° of visual angle. The eye tracker was controlled by the iView X software (SMI iView X, SensoMotoric Instruments, Tetow, Germany) and fixated to the presentation monitor with a display mode of 1680 × 1050 pixels. Subjects were placed ~65 cm in front of the monitor, while the position of their left eye was sampled at 120 Hz. Calibration was performed following the built-in 9-point procedure at the beginning of the experiments.

#### Preprocessing of Eye Tracking Data

One subject reported to have misunderstood the instructions not to focus on the visual areas outside the circle and was therefore excluded from any further analyses. For the remaining subjects, fixation detection was done following the same pipeline as described in Experiment 1. Since there was no evidence for slow, drift-like displacements of the recorded fixation coordinates, no correction was applied. No subjects (due to calibration outliers or no valid trials) and no trials (due to only one fixation detected) were excluded using the outlier criteria of Experiment 1. To identify possible deviations from the viewing instructions per trial, the theoretically expected fixation pattern, given by the coordinates of the moving circle at each time point, was correlated to the actual fixation pattern measured by eye tracking. One subject had a correlation below the outlier threshold (defined analogously to Experiment 1) for more than one-third of all trials and was therefore excluded. Further analyses of the free recall performance were therefore based on a total of 64 subjects (32 females; mean age = 23.28, SD = 3.94, range 18–32).

#### Experimental Manipulation of Visual Exploration

The influence of the three experimental conditions on memory performance was assessed in R with a linear mixed model (“nlme” package, Pinheiro et al. 2019) combined with ANOVA (SS II). The participant ID was included as the random effect in the model. The dependent variable was the free recall performance. The independent variable was the factor “experimental condition.” We tested for the main effect of the experimental condition while controlling for sex and age. Post hoc tests were applied to further investigate the effect, and *P* values were FDR-corrected by the number of post hoc tests conducted (=2, corresponding to the comparison of the “Guided Fixation” condition with the “Area of no Interest” condition and with the “Guided Fixation × 2” condition, respectively). Effect sizes for repeated measures are indicated by generalized semipartial *R*^2^β^*^ (Jaeger et al. 2017).

## Results

### Experiment 1

In this experiment, subjects viewed 72 photographs of complex scenes with different emotional valences (i.e., neutral, positive, and negative scenes) and 24 scrambled pictures in the fMRI scanner, followed by a free recall test outside of the scanner.

#### Fixations—Descriptive Statistics

On average, subjects made 5.30 fixations per picture (SD = 1.01; mean duration 459.39 ms, SD = 126.01 ms) with a mean distance of 7.82° (visual angle; SD = 1.22) between two subsequent fixations. An average of 3.51 (SD = 0.94) fixations were lying inside any AOI of a picture, covering a total of 2.45 unique AOIs (54%, SD = 0.52). Blinks occurred in 26% of all trials and covered 8% (*M =* 206.85 ms, SD = 186.95 ms) of a picture trial on average.

#### Visual Exploration and Memory

On the behavioral level, we first investigated the association between several visual exploration characteristics and subsequent memory performance (for descriptive statistics, see Supplementary Results).

For the variable of primary interest (i.e., the number of fixations), we found a positive correlation with free recall performance (*t*(1351) = 3.70, *P* = 3.1e−04, *R*^2^β^*^ = 0.011, 95% CI [0.004, 0.021]). The interaction of the number of fixations with emotional picture valence was not significant (*F*(2, 1351) = 1.36, *P* = 0.83). Because memory performance might rely on the sampling of specific regions of each picture, fixations were subsequently restricted to areas of interest (AOIs), which covered semantically informative areas (Holmqvist et al. 2011). In our experiment, the fixation frequency in AOIs was highly correlated with the fixation frequency in the entire picture (*r* = 0.82).

There was a significant positive correlation of the number of fixations in AOIs with free recall performance (*t*(1336) = 5.34, *P* = 4.4e−07, *R*^2^β^*^ = 0.021, 95% CI [0.011, 0.035]). The interaction with valence was not significant (*F*(2, 1336) = 0.19, *P* = 0.83). Within the variables of secondary interest, blink duration was negatively associated with free recall performance (*t*(1303) = −4.46, *P* = 1.8e−05, *R*^2^β^*^ = 0.014, 95% CI [0.006, 0.026]) without a significant valence interaction (*F*(2, 1303) = 0.36, *P* = 0.83). No association was found for the average distance between sequential fixations and free recall performance (*t*(1351) = −1.43, *P* = 0.15); for results regarding the recognition performance, see Supplementary Table 3.

The positive link between sampling frequency in semantically informative regions and memory performance is in line with previous literature and extends this finding with regard to the type of information processed (i.e., complex scenes) and type of memory tested (i.e., free recall, a purely episodic form of memory).

#### Fixations and fMRI

Next, we examined the relationship between fixation frequency in semantically informative regions and functional brain activation (independent of memory). The linear parametric modulation effect of the number of fixations in AOIs on the fMRI signal was investigated for the contrast “viewing real pictures versus viewing scrambled pictures” throughout the brain (*N*_voxels_ =  57 032).

**Figure 4:**
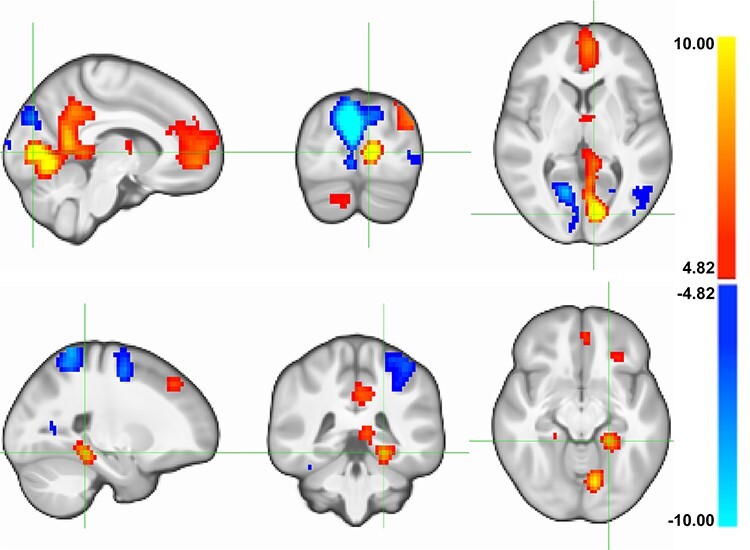
Association between the number of fixations in AOIs and the fMRI-encoding signal. Parametric modulation effect of the number of fixations in AOIs on the fMRI-encoding signal in 775 subjects, for every voxel of the brain (*N*_voxels_ = 57 032) for the contrast between viewing real pictures and scrambled pictures (*p*_FWE-WB_ < 0.05 corresponding to *t*(771) ≥/≤ ±4.82). Top: focus on the large cluster located in the left pericalcarine gyrus, extending to left lingual areas and bilaterally to precuneus and cingulum. Bottom: focus on the cluster located in the left MTL. FWE, family-wise error; WB, whole-brain correction for the investigated number of voxels in brain.

A positive modulation of brain activation by the fixation frequency was found in two large clusters, both being located predominantly in the medial left hemisphere. One was located in the left pericalcarine gyrus, extending to left lingual areas and bilaterally to precuneus and cingulum. The other was found in the left orbitofrontal cortex, including parts of the anterior cingulate and the bilateral superior frontal cortices. Additional clusters comprised bilateral parts of the MTL and thalamus as well as the left inferior parietal cortex and the right cerebellum. Negative modulation of brain activation by the fixation frequency comprised a major cluster in the right cuneus, reaching into right lingual areas and bilateral parts of the superior parietal cortex (see Fig. 4 and Supplementary Table 1).

To summarize, a positive relationship between fixations and the fMRI signal was identified in early perceptual processing (e.g., left lingual) regions and regions known to be related to successful memory encoding, including the MTL.

#### fMRI and Memory

**Figure 5:**
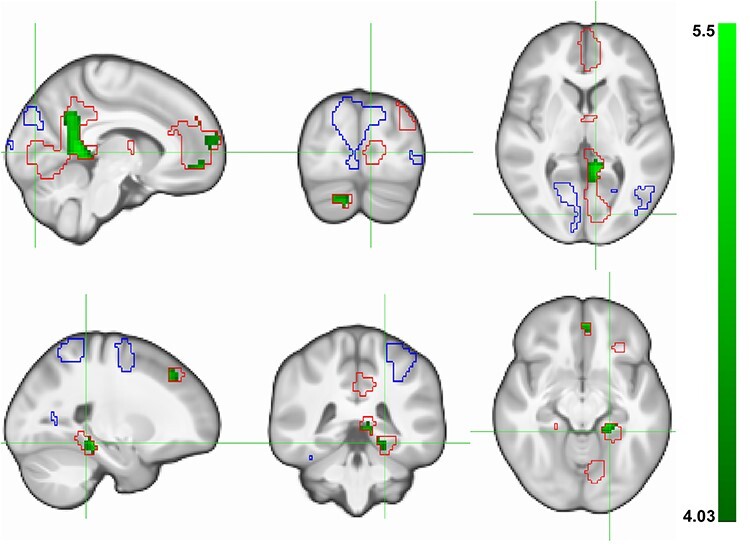
Association between the fMRI-encoding signal and episodic memory performance. Association between the fMRI signal at encoding and subsequent free recall in 1395 subjects, restricted to voxels showing either a positive (red outline) or negative (blue outline) activation related to the number of fixations in AOIs (*p*_FWE-SVC_ < 0.05 corresponding to *t*(1388) ≥/≤ ±4.03). Top: focus on the large cluster located in the left pericalcarine gyrus, extending to left lingual areas and bilaterally to precuneus and cingulum. Bottom: focus on the cluster located in the left MTL. FWE, family-wise error; SVC, small volume correction for the number of voxels in outlined regions.

We then determined if brain activation in the previously detected fixations-related clusters is related to memory performance. Therefore, only voxels showing either a positive or a negative modulation of activity by the number of fixations in AOIs were considered. The associations between the fMRI-encoding signal and free recall were investigated in those voxels, revealing clusters of the right cerebellar cortex, the left superior frontal cortex, and the left parahippocampal gyrus (see Fig. 5 and Supplementary Table 1). Importantly, the analyses revealed only positive associations, exclusively and consistently found in clusters with positive parametric modulation effects. We thereby show that activity in the reported regions was associated with a great extent with free recall performance.

It is important to note that the behavioral and imaging findings reported so far are of correlational nature. Although our and previous results are in line with the idea that sampling intensity affects memory performance, they do not speak of causation. At this stage we cannot rule out that there is no causal relation or that the causal direction is inversed, meaning that a good memory in general positively affects sampling frequency. In fact, there is evidence suggesting that previous experiences might influence visual exploration, including the number of fixations (Sharot et al. 2008; Hannula 2010; Wolfe and Horowitz 2017; Lancry-Dayan et al. 2019).

Furthermore, in the present experimental setting, the number of fixations and the number of AOIs covered by them were highly correlated. It is therefore impossible to distinguish between the importance of the frequency and location of such fixations for subsequent memory performance. We cannot preclude that the increased amount of gathered semantic information, rather than the sampling intensity per se, is related to successful memory processing.

To address these questions, we conducted an additional experiment predefining the visual scan paths for 64 subjects during picture encoding. The first aim was to examine the causal link between exploration characteristics and memory performance under this experimental manipulation. The second aim was to separately investigate the effects of the number of fixations and the number of AOIs covered by them on memory performance.

### Experiment 2

In this experiment, subjects viewed 54 photographs of complex scenes with different emotional valences (i.e., neutral, positive, and negative scenes) under guided viewing conditions, followed by a free recall test.

#### Scan Path—Descriptive Statistics

The mean correlation between the scan path of the moving circle and the actual fixation pattern was high (*r =* 0.87), with only a small time lag between the moving circle arriving at a new picture region and the first fixation within this region (*M =* 74.05 ms, SD = 66.29), indicating good compliance and accuracy. Furthermore, the empirically measured fixations per picture indicated that the fixation frequencies in the “Area of no Interest” (*M =* 5.81, SD = 0.92) and the “Guided Fixation” condition were similar (*M =* 5.59, SD = 0.94) while increased in the “Guided Fixation × 2” condition (*M =* 10.24, SD = 2.23; see Supplementary Fig. 1). The empirically measured durations of fixations per picture in the “Area of no Interest” (*M =* 1047.18 ms, SD = 451.61 ms) and the “Guided Fixation” condition were again similar (*M =* 1162.10 ms, SD = 479.67 ms) while decreased in the “Guided Fixation × 2” condition (*M =* 692.54 ms, SD = 227.08 ms).

#### Visual Exploration and Memory

To study the effects of fixation frequency and location (i.e., the number of fixations in AOIs and the number of AOIs covered, respectively) on episodic memory performance in isolation, we introduced three within-subject experimental conditions as described in Figure 3. The task was to encode the pictures following the path of a moving circle. The “Area of no Interest” and “Guided Fixation” condition only differed with regard to the number of AOIs (i.e., increased in the “Guided Fixation” condition). The “Guided Fixation” and the “Guided Fixation × 2” conditions only differed in the number of fixations (i.e., doubled in the “Guided Fixation × 2” condition) in the AOIs.

**Figure 6:**
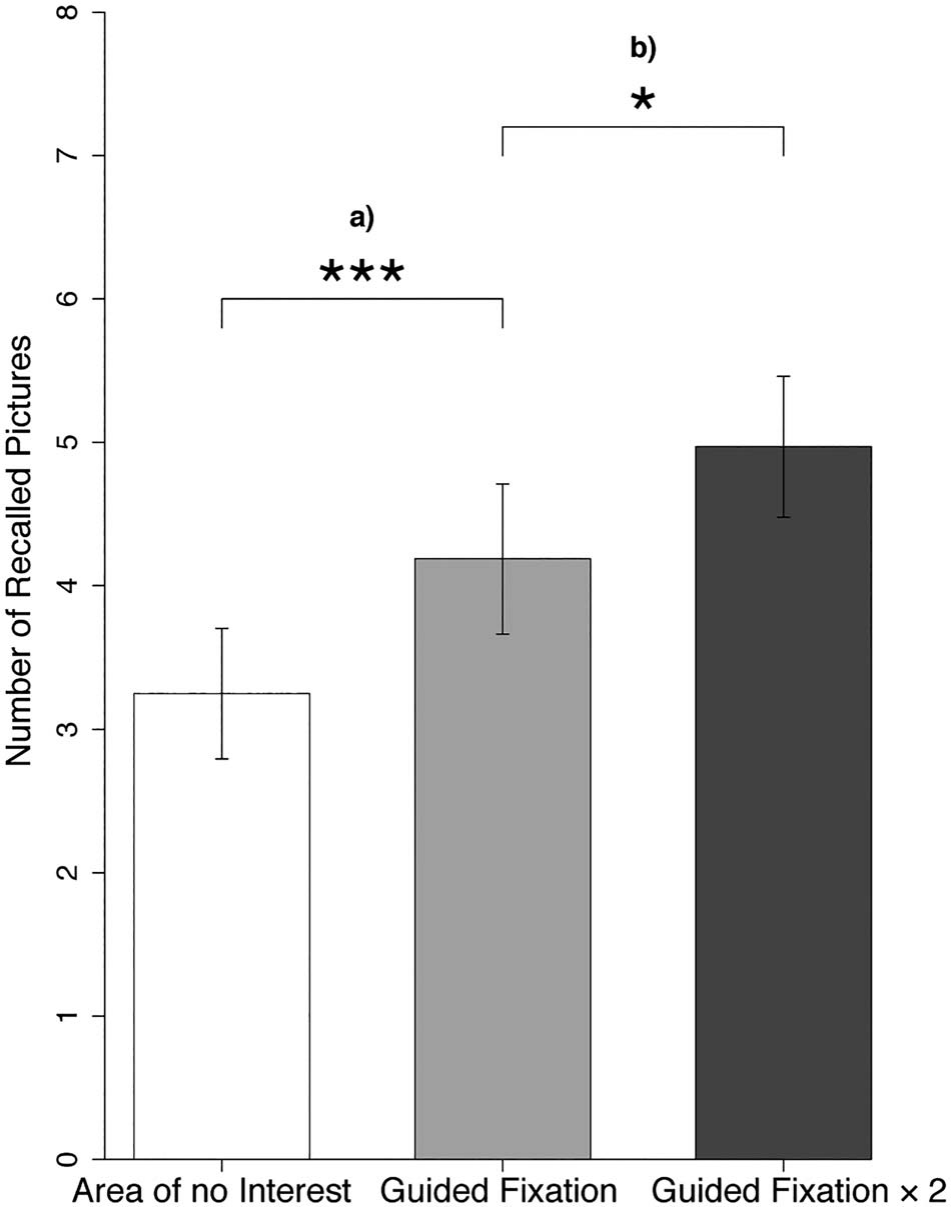
Effect of scan path manipulation on free recall performance. Episodic memory effect in 64 subjects by (a) decreasing the number of AOIs covered by fixations, leading to a lower amount of freely recalled pictures in the “Area of no Interest” condition (*M =* 3.25, SE = 0.46) compared with the “Guided Fixation” condition (*M =* 4.19, SE = 0.52) as well as by (b) increasing fixation frequency, leading to a higher free recall performance in the “Guided Fixation × 2” condition (*M =* 4.97, SE = 0.49) compared with the “Guided Fixation” condition.

For free recall performance, there was a positive main effect of the factor “experimental condition” (*F*(2, 126) = 20.70, *P* = 1.7e−08). Post hoc tests revealed an average decrease of freely recalled pictures in the “Area of no Interest” condition compared with the “Guided Fixation” condition by 22% (*t*(63) = −3.79, *P* = 2.9e−04, *R*^2^β^*^ = 0.063, 95% CI [0.007, 0.165]), as well as an average increase of freely recalled pictures in the “Guided Fixation × 2” condition compared with the “Guided Fixation” condition by 19% (*t*(63) = 2.51, *P* = 0.015, *R*^2^β^*^ = 0.036, 95% CI [0.001, 0.122]; Fig. 6).

## Discussion

Experiment 1 revealed a link between visual exploration and memory. Most importantly, there was a positive correlation of the number of fixations in semantically informative picture areas and the performance in the subsequent episodic memory task. Fixations allow for the extraction and processing of detailed information like the position or orientation of objects (Pertzov et al. 2009). Episodic memory is typically characterized by the ability to recall such details.

Further, we found a negative correlation between blink duration and episodic memory performance. A possible explanation is that during blinks, which might reflect sleepiness, no visual sampling is taking place, and hence, less information is encoded. Finally, we did not find a correlation between the interfixation distance and episodic memory performance, indicating that this exploration characteristic per se is not affecting memory strength.

The neuroimaging data of Experiment 1 revealed that the number of fixations in semantically informative picture areas was correlated with brain activation in regions important for vision and memory. The activated brain regions overlap with the parietal medial temporal pathway, which is associated with target-directed fixations in animals (Kravitz et al. 2011) and includes the MTL. Activation of the MTL has been repeatedly and consistently related to successful memory encoding in human neuroimaging studies (Dickerson and Eichenbaum 2010). Growing evidence additionally attributes to the MTL a critical role in perceptual processing (Ringo et al. 1994; Hodgetts et al. 2017; Dalton et al. 2018; Ryan et al. 2020). Our findings further suggest the recruitment of frontocerebellar circuits. They are implicated in strategic planning of subsequent eye movements (Voss, Gonsalves, et al. 2011a; Voss, Warren, et al. 2011b), have been previously found to be activated in visual episodic memory tasks (Niu et al. 2012), and could be functionally connected to the MTL via the thalamus (Ito et al. 2015). Interestingly with regard to the MTL, Experiment 1 has predominantly shown an association between memory performance and activation in the parahippocampal gyrus, while an earlier study using a face recognition paradigm identified the hippocampus to be most critically involved in both in visual sampling and memory formation (Liu et al. 2017). This discrepancy could be interpreted in the light of the “binding of item and context” model (Diana et al. 2007). The model proposes that both spatial and nonspatial contextual information is stored by the parahippocampal gyrus, while the hippocampus binds this information, integrating additional item information provided by the perirhinal cortex (Graham et al. 2010). We argue that for complex scene viewing, as opposed to faces, the picture context (e.g., the number and strength of the contextual associations within a scene) and the temporal context (e.g., the order of scenes when trying to memorize them as a story) are particularly important (Bar et al. 2008). In addition, due to the heterogeneity of the scenes used, it might be sufficient to recall some of them based on a serial, single feature identification strategy without rich associations between them, which would require less hippocampus involvement (Graham et al. 2010). This might partly explain why the effect size for the association between fixation frequency and memory in Experiment 1 (*R*^2^β^*^ = 0.011) was smaller than reported by Olsen et al. (2016) in a face recognition paradigm (*R*^2^ = 0.11).

In Experiment 2, we found that manipulating the scan path affects subsequent memory performance. As anticipated, visual exploration in semantically unimportant picture regions, as compared with regions with more semantic information, decreased subsequent free recall performance by 22%. This is in line with earlier findings indicating that restricted focused visual input decreases subsequent memory (Pertzov et al. 2009; Damiano and Walther 2019). More importantly, however, Experiment 2 is the first to our knowledge to show a memory effect by repeating focused visual input that was otherwise held constant. Doubling the number of fixations in semantically important regions within a given exploration time increased subsequent free recall performance by 19%. Since guided viewing might per se interfere with memory encoding (Voss, Gonsalves, et al. 2011a; Voss, Warren, et al. 2011b), it remains to be investigated how this finding translates to free viewing. However, the evidence for causality and direction of the relationship between number of fixations and recall performance enabled further interpretation of the imaging results of Experiment 1. Specifically, the findings are in line with the idea that visual sampling frequency triggers not only visual regions but a larger brain circuitry relevant for memory processing, including the MTL. What caused individuals to scan scenes at varying sampling frequency in the first place has to be further examined. One explanation might be different levels of expertise. Several studies have associated expert knowledge to altered scanning of expertise-related objects or scenes. Interestingly, more fixations and more dwells in AOIs were specifically identified as key features in experts that could be responsible for their superior performance on perceptual–cognitive tasks (Gegenfurtner et al. 2011; Brams et al. 2019). Due to the variety of the scenes used in both experiments, it seems implausible that some of the subjects had specific expertise for the scenes presented. However, it has been argued that very short-term relational memory signals, provided by the MTL, are needed for effective visual episodic memory formation, for example, to bind together important visual features over space and time (Liu et al. 2017; Voss et al. 2017). As such, effective MTL signaling is likely to be not only a consequence of visual scanning but also its prerequisite (Ryan et al. 2020).

Our results have several important implications. First, they suggest the importance of measuring eye movements in visual memory studies. A similar claim has already been put forward by Voss et al. (2017), arguing that visual exploration systematically covaries with cognitive variables of interest such as attention, emotion, and intentionality, thereby confounding their effect on memory mechanisms. We add the notion that the frequency and location of fixations are cognitive variables of interest by themselves. They vary across individuals, are associated with brain activations in memory-related regions, affect episodic memory formation, and should thus be considered as an integrative part of memory processing.

Second, our findings may partly explain memory deficits in neuropsychiatric conditions and open a new treatment approach. Both memory deficits and altered exploration patterns are often observed in neuropsychiatric conditions, such as depression (Kellough et al. 2008; Elliott et al. 2011), dementia (Shakespeare et al. 2015), anxiety disorders (LeMoult and Joormann 2012), autism spectrum disorders (Fedor et al. 2018), posttraumatic stress disorder (Armstrong et al. 2013; de Quervain et al. 2017), and schizophrenia (Williams et al. 2010). Patients with schizophrenia, for example, have difficulties executing simple visual tasks like smooth pursuit (O’Driscoll and Callahan 2008). They also show less fixations and visual exploration of semantically complex pictures (Beedie 2011). Therefore, a training aimed at increasing fixations during visual exploration might prove useful for enhancing memory in conditions of impaired memory functions. In summary, our data indicate that higher fixation frequency improves visual memory, a phenomenon with great therapeutic potential.

## Notes

The data that support the findings of this study are available from the corresponding author on request. Custom code that supports the findings of this study is publicly available on OSF (https://osf.io/r9bxd/).

## Funding

Transfaculty Research Platform Molecular and Cognitive Neurosciences, University of Basel, 4055 Basel, Switzerland; the Swiss National Science Foundation (P0BSP1_168917 to B.F.). *Conflict of Interest*: None declared.

## Supplementary Material

supplementary_material_clean_tgaa032

## References

[ref1] Armstrong T , Bilsky SA, Zhao M, Olatunji BO. 2013. Dwelling on potential threat cues: an eye movement marker for combat related PTSD. Depress Anxiety. 30(5):497–502. 23620193 10.1002/da.22115

[ref2] Ashburner J . 2007. A fast diffeomorphic image registration algorithm. NeuroImage. 38(1):95–113. 17761438 10.1016/j.neuroimage.2007.07.007

[ref3] Bar M , Aminoff E, Schacter DL. 2008. Scenes unseen: the parahippocampal cortex intrinsically subserves contextual associations, not scenes or places per se. J Neurosci. 28(34):8539–8544. 18716212 10.1523/JNEUROSCI.0987-08.2008 PMC2707255

[ref4] Beedie S . 2011. Atypical scanpaths in schizophrenia: evidence of a trait- or state-dependent phenomenon? J Psychiatry Neurosci. 36(3):150–164. 21223647 10.1503/jpn.090169 PMC3080511

[ref5] Boghen D , Troost BT, Daroff RB, Dell’Osso LF, Birkett JE. 1974. Velocity characteristics of normal human saccades. Investig Ophthalmol. 13(8):619–623. 4841869

[ref6] Brams S , Ziv G, Levin O, Spitz J, Wagemans J, Williams AM, Helsen WF. 2019. The relationship between gaze behavior, expertise, and performance: a systematic review. Psychol Bull. 145(10):980–1027. 31414844 10.1037/bul0000207

[ref7] Bylinskii Z , Isola P, Bainbridge C, Torralba A, Oliva A. 2015. Intrinsic and extrinsic effects on image memorability. Vis Res. 116:165–178. 25796976 10.1016/j.visres.2015.03.005

[ref8] Ciollaro M , Wang D. 2016. MeanShift: clustering via the mean shift algorithm. The Comprehensive R Archive Network. https://CRAN.R-project.org/package=MeanShift.

[ref9] Dalton MA , Zeidman P, McCormick C, Maguire EA. 2018. Differentiable processing of objects, associations, and scenes within the hippocampus. J Neurosci. 38(38):8146–8159. 30082418 10.1523/JNEUROSCI.0263-18.2018 PMC6146500

[ref10] Damiano C , Walther DB. 2019. Distinct roles of eye movements during memory encoding and retrieval. Cognition. 184:119–129. 30594878 10.1016/j.cognition.2018.12.014

[ref43] de Quervain D , Schwabe L, Roozendaal B. 2017. Stress, glucocorticoids and memory: implications for treating fear-related disorders. Nat Rev Neurosci. 18(1):7–19. 27881856 10.1038/nrn.2016.155

[ref11] Diana RA , Yonelinas AP, Ranganath C. 2007. Imaging recollection and familiarity in the medial temporal lobe: a three-component model. Trends Cogn Sci. 11(9):379–386. 17707683 10.1016/j.tics.2007.08.001

[ref12] Dickerson BC , Eichenbaum H. 2010. The episodic memory system: neurocircuitry and disorders. Neuropsychopharmacology. 35(1):86–104. 19776728 10.1038/npp.2009.126 PMC2882963

[ref13] Elliott R , Zahn R, Deakin JFW, Anderson IM. 2011. Affective cognition and its disruption in mood disorders. Neuropsychopharmacology. 36(1):153–182. 20571485 10.1038/npp.2010.77 PMC3055516

[ref14] Fedor J , Lynn A, Foran W, DiCicco-Bloom J, Luna B, O’Hearn K. 2018. Patterns of fixation during face recognition: differences in autism across age. Autism. 22(7):866–880. 28782371 10.1177/1362361317714989 PMC6599607

[ref15] Gegenfurtner A , Lehtinen E, Säljö R. 2011. Expertise differences in the comprehension of visualizations: a meta-analysis of eye-tracking research in professional domains. Educ Psychol Rev. 23(4):523–552.

[ref16] Graham KS , Barense MD, Lee ACH. 2010. Going beyond LTM in the MTL: a synthesis of neuropsychological and neuroimaging findings on the role of the medial temporal lobe in memory and perception. Neuropsychologia. 48(4):831–853. 20074580 10.1016/j.neuropsychologia.2010.01.001

[ref17] Hannula DE . 2010. Worth a glance: using eye movements to investigate the cognitive neuroscience of memory. Front Hum Neurosci. 4:166. 21151363 10.3389/fnhum.2010.00166 PMC2995997

[ref18] Heck A , Fastenrath M, Ackermann S, Auschra B, Bickel H, Coynel D, Gschwind L, Jessen F, Kaduszkiewicz H, Maier W, et al. 2014. Converging genetic and functional brain imaging evidence links neuronal excitability to working memory, psychiatric disease, and brain activity. Neuron. 81(5):1203–1213. 24529980 10.1016/j.neuron.2014.01.010 PMC4205276

[ref19] Heisz JJ , Pottruff MM, Shore DI. 2013. Females scan more than males: a potential mechanism for sex differences in recognition memory. Psychol Sci. 24(7):1157–1163. 23696202 10.1177/0956797612468281

[ref20] Henderson J . 2003. Human gaze control during real-world scene perception. Trends Cogn Sci. 7(11):498–504. 14585447 10.1016/j.tics.2003.09.006

[ref21] Henderson JM , Hayes TR. 2017. Meaning-based guidance of attention in scenes as revealed by meaning maps. Nat Hum Behav. 1(10):743. 31024101 10.1038/s41562-017-0208-0 PMC7455012

[ref22] Hodgetts CJ , Voets NL, Thomas AG, Clare S, Lawrence AD, Graham KS. 2017. Ultra-high-field fMRI reveals a role for the Subiculum in scene perceptual discrimination. J Neurosci. 37(12):3150–3159. 28213445 10.1523/JNEUROSCI.3225-16.2017 PMC5373110

[ref23] Holmqvist K , Nyström M, Andersson R, Dewhurst R, Jarodzka H, Van de Weijer J. 2011. Areas of interest. In: Eye tracking: a comprehensive guide to methods and measures. Oxford (NY): Oxford University Press.

[ref24] Ito HT , Zhang S-J, Witter MP, Moser EI, Moser M-B. 2015. A prefrontal-thalamo-hippocampal circuit for goal-directed spatial navigation. Nature. 522(7554):50–55. 26017312 10.1038/nature14396

[ref25] Itti L , Koch C. 2001. Computational modelling of visual attention. Nat Rev Neurosci. 2(3):194–203. 11256080 10.1038/35058500

[ref26] Jaeger BC , Edwards LJ, Das K, Sen PK. 2017. An R 2 statistic for fixed effects in the generalized linear mixed model. J Appl Stat. 44(6):1086–1105.

[ref27] Jenkinson M , Beckmann CF, Behrens TEJ, Woolrich MW, Smith SM. 2012. FSL. NeuroImage. 62(2):782–790. 21979382 10.1016/j.neuroimage.2011.09.015

[ref28] Kafkas A , Montaldi D. 2011. Recognition memory strength is predicted by pupillary responses at encoding while fixation patterns distinguish recollection from familiarity. Q J Exp Psychol. 64(10):1971–1989. 10.1080/17470218.2011.588335 21838656

[ref29] Kellough JL , Beevers CG, Ellis AJ, Wells TT. 2008. Time course of selective attention in clinically depressed young adults: an eye tracking study. Behav Res Ther. 46(11):1238–1243. 18760771 10.1016/j.brat.2008.07.004 PMC2584153

[ref30] Klein A , Andersson J, Ardekani BA, Ashburner J, Avants B, Chiang M-C, Christensen GE, Collins DL, Gee J, Hellier P, et al. 2009. Evaluation of 14 nonlinear deformation algorithms applied to human brain MRI registration. NeuroImage. 46(3):786–802. 19195496 10.1016/j.neuroimage.2008.12.037 PMC2747506

[ref31] Kravitz DJ , Saleem KS, Baker CI, Mishkin M. 2011. A new neural framework for visuospatial processing. Nat Rev Neurosci. 12(4):217–230. 21415848 10.1038/nrn3008 PMC3388718

[ref32] Lancry-Dayan OC , Kupershmidt G, Pertzov Y. 2019. Been there, seen that, done that: modification of visual exploration across repeated exposures. J Vis. 19(12):2. 10.1167/19.12.2 31585463

[ref33] Lang PJ , Bradley MM, Cuthbert BN. 2008. International Affective Picture System (IAPS): affective ratings of pictures and instruction manual. Gainesville, FL: University of Florida.

[ref34] LeMoult J , Joormann J. 2012. Attention and memory biases in social anxiety disorder: the role of comorbid depression. Cogn Ther Res. 36(1):47–57. 10.1007/s10608-010-9322-2 PMC3475322 23087492

[ref35] Liu Z-X , Shen K, Olsen RK, Ryan JD. 2017. Visual sampling predicts hippocampal activity. J Neurosci. 37(3):599–609. 28100742 10.1523/JNEUROSCI.2610-16.2016 PMC6596763

[ref36] McCabe DP , Geraci L, Boman JK, Sensenig AE, Rhodes MG. 2011. On the validity of remember–know judgments: evidence from think aloud protocols. Conscious Cogn. 20(4):1625–1633. 21963257 10.1016/j.concog.2011.08.012

[ref37] Nakagawa S , Schielzeth H. 2013. A general and simple method for obtaining R 2 from generalized linear mixed-effects models. Methods Ecol Evol. 4(2):133–142.

[ref38] Niu Y , Todd RM, Anderson AL. 2012. Affective salience can reverse the effects of stimulus-driven salience on eye movements in complex scenes. Front Psychol. 3:336. 23055990 10.3389/fpsyg.2012.00336 PMC3457078

[ref39] O’Driscoll GA , Callahan BL. 2008. Smooth pursuit in schizophrenia: a meta-analytic review of research since 1993. Brain Cogn. 68(3):359–370. 18845372 10.1016/j.bandc.2008.08.023

[ref40] Olsen RK , Sebanayagam V, Lee Y, Moscovitch M, Grady CL, Rosenbaum RS, Ryan JD. 2016. The relationship between eye movements and subsequent recognition: evidence from individual differences and amnesia. Cortex. 85:182–193. 27842701 10.1016/j.cortex.2016.10.007

[ref41] Pertzov Y , Avidan G, Zohary E. 2009. Accumulation of visual information across multiple fixations. J Vis. 9(10):2–2. 10.1167/9.10.2 19810783

[ref42] Pinheiro J , Bates D, R-core. 2019. nlme: linear and nonlinear mixed effects models. https://CRAN.R-project.org/package=nlme.

[ref44] Ringo JL , Sobotka S, Diltz MD, Bunce CM. 1994. Eye movements modulate activity in hippocampal, parahippocampal, and inferotemporal neurons. J Neurophysiol. 71(3):1285–1288. 8201422 10.1152/jn.1994.71.3.1285

[ref45] Ross J , Morrone MC, Goldberg ME, Burr DC. 2001. Changes in visual perception at the time of saccades. Trends Neurosci. 24(2):113–121. 11164942 10.1016/s0166-2236(00)01685-4

[ref46] Ryan JD , Shen K, Liu Z-X. 2020. The intersection between the oculomotor and hippocampal memory systems: empirical developments and clinical implications. Ann N Y Acad Sci. 1464(1):115–141. 31617589 10.1111/nyas.14256 PMC7154681

[ref47] Shakespeare TJ , Pertzov Y, Yong KXX, Nicholas J, Crutch SJ. 2015. Reduced modulation of scanpaths in response to task demands in posterior cortical atrophy. Neuropsychologia. 68:190–200. 25596316 10.1016/j.neuropsychologia.2015.01.020

[ref48] Sharot T , Davidson ML, Carson MM, Phelps EA. 2008. Eye movements predict recollective experience. Lauwereyns J, editor. PLoS One. 3(8):e2884. 18682731 10.1371/journal.pone.0002884 PMC2478711

[ref49] Smith SW . 2003. Digital signal processing: a practical guide for engineers and scientists. Amsterdam, Boston (MA): Newnes.

[ref50] Van Orden KF , Jung T-P, Makeig S. 2000. Combined eye activity measures accurately estimate changes in sustained visual task performance. Biol Psychol. 52(3):221–240. 10725565 10.1016/s0301-0511(99)00043-5

[ref51] von der Malsburg T . 2015. Saccades: detection of fixations in eye-tracking data. The Comprehensive R Archive Network. https://CRAN.R-project.org/package=saccades.

[ref52] Voss JL , Bridge DJ, Cohen NJ, Walker JA. 2017. A closer look at the hippocampus and memory. Trends Cogn Sci. 21(8):577–588. 28625353 10.1016/j.tics.2017.05.008 PMC5659202

[ref53] Voss JL , Gonsalves BD, Federmeier KD, Tranel D, Cohen NJ. 2011a. Hippocampal brain-network coordination during volitional exploratory behavior enhances learning. Nat Neurosci. 14(1):115–120. 21102449 10.1038/nn.2693 PMC3057495

[ref54] Voss JL , Warren DE, Gonsalves BD, Federmeier KD, Tranel D, Cohen NJ. 2011b. Spontaneous revisitation during visual exploration as a link among strategic behavior, learning, and the hippocampus. Proc Natl Acad Sci. 108(31):E402–E409. 21768385 10.1073/pnas.1100225108 PMC3150890

[ref55] Wilcox RR . 2012. Modern statistics for the social and behavioral sciences: a practical introduction. Boca Raton (FL): Taylor & Francis.

[ref56] Williams LE , Must A, Avery S, Woolard A, Woodward ND, Cohen NJ, Heckers S. 2010. Eye-movement behavior reveals relational memory impairment in schizophrenia. Biol Psychiatry. 68(7):617–624. 20655509 10.1016/j.biopsych.2010.05.035 PMC3184178

[ref57] Wixted JT . 2009. Remember/know judgments in cognitive neuroscience: an illustration of the underrepresented point of view. Learn Mem. 16(7):406–412. 19546229 10.1101/lm.1312809

[ref58] Wolfe JM , Horowitz TS. 2017. Five factors that guide attention in visual search. Nat Hum Behav. 1(3):0058. 10.1038/s41562-017-0058 PMC9879335 36711068

