# Supplementary material for: Visual Exploration at Higher Fixation Frequency Increases Subsequent Memory Recall
Source: Cereb Cortex Commun. 2020 Jul 21;1(1):tgaa032. doi: 10.1093/texcom/tgaa032 (PMC8153053; doi:10.1093/texcom/tgaa032)
Supplement: supplementary_material_clean_tgaa032 [file supplementary_material_clean_tgaa032.docx]

# Supplementary Materials and Methods Experiment 1

## Recognition task

For the recognition task, an additional 72 pictures were taken from the same database and matched to the encoding set in terms of valence category and semantic content (24 pictures per category). This resulted in a total of 144 pictures for the recognition task, half of which were old (i.e. presented during the encoding task), the other half being new (i.e. not presented before).

Each of the 144 recognition trials started with a fixation cross, presented for 500 ms against a dark background, and was followed by the presentation of one picture for 1 s. The stimulus onset time was jittered within 3 s (1 TR) per valence and old/new category with regard to the scan onset. A blank, dark screen followed the offset of the picture for 1 s. Afterwards, participants subjectively rated the picture as remembered, familiar, or new by button press. Picture rating was possible in a time window of 3 s. Across all trials, pictures were presented in a quasi-randomized order, allowing for a maximum of 4 consecutive pictures with identical valence categories. Recognition performance was assessed by the difference between subsequently remembered and subsequently not remembered pictures (Luksys et al. 2015).

We assessed the relationship between recognition performance and eye tracking parameters as well as the fMRI signal associated with the number of fixations at encoding. Therefore, the models specified for the free recall were used (see Materials and Methods Experiment 1), but with the recognition performance as the dependent variable. Results are summarized in the Supplementary Tables S1 and S3.

## Construction of ET-AOIs

Fixations of 200 subjects were randomly chosen per picture. All fixations are iteratively and simultaneously moved towards locations of higher spatial density until points of eventual convergence. The points represent the modes of the distribution and indicate the distinct clusters identified. In order to build AOIs, large clusters (containing a minimum of 2.5% of the fixations) were given a parametrized representation as covariance ellipses. The ellipses are centered at the cluster mean (centroid) and represent 50% of the spatial variance of the original cluster. The procedure is based on previous work (Santella and DeCarlo 2004), with a modified threshold for spatial variance to prevent overlapping clusters. To estimate the reliability of this approach, the same procedure was then repeated 100 times per picture. For each iteration, the spatial overlap of the resulting AOIs with the initial solution was calculated by the Sørensen-Dice coefficient, ranging from 0 to 1 for non-overlapping and perfectly identical AOIs, respectively (Dice 1945). The mean across all 72 pictures and 100 repetitions was .93 (*SD* = .03), suggesting a high reliability of the AOI estimation.

## Population-average anatomical probabilistic atlas

The T1-weighted image of each subject of the 1000 subjects included in the construction of the study-specific template was automatically segmented into cortical and subcortical structures using FreeSurfer (v4.5, RRID:SCR_001847; http://surfer.nmr.mgh.harvard.edu/, Fischl et al. 2002). On the basis of the Desikan-Killiany-atlas (Desikan et al. 2006), 35 cortical gyri were labeled, as well as 17 subcortical regions (see Fischl et al. 2002). The segmented T1 images were then normalized to the study-specific anatomical template. Finally, the normalized segmentations were averaged across subjects, resulting in a population-average probabilistic atlas. Every voxel of the template could consequently be assigned a probability of belonging to a given anatomical structure.

## Grand average pupil profile

The grand average pupil profile is based on 800 subjects (482 females; mean age = 22.34, *SD* = 3.38, range 18–35) with eye tracking data that were not identified as outliers for calibration data or the eye movement velocity distribution. Eye-blink related artifacts were replaced by linear interpolation throughout the dataset. Pupil data were then smoothed using a five-point unweighted average filter applied twice (Siegle et al. 2003) and segmented per trial. The segmentation window spanned a total of 3 seconds, including the 500 ms before picture onset that served as a baseline. For each trial with a minimum of 2 fixations during picture presentation, pupil data (recorded in arbitrary units) were baseline-corrected. The correction value was computed as a one-step M-estimator of location of the corresponding 30 pupil height samples (’WRS2’ package, Mair and Wilcox 2019). This procedure is a proposed alternative to mean averaging that is more robust against outliers (Wilcox 2012). Valid trials, each consisting of a time series of 150 corrected pupil height samples that describe the pupil dynamics during the 2.5 s of picture presentation, formed the basis for further aggregation. This was done in two steps, again using the one-step estimator. First, for each subject and time point separately, trials were averaged across valence categories. Second, the resulting pupil profiles were averaged across subjects, resulting in the grand average pupil profile. The pupil profile of each trial was separately correlated with this grand average. A low correlation led to trial exclusion, with the cutoff-threshold (*r* < .75) based on boxplots and ideal fourths used for quartile estimation (Wilcox 2012).

# Supplementary Materials and Methods Experiment 2

## Recognition task

The recognition task is almost identical to the one used in experiment 1, with the exception of the stimulus onset time not being jittered. The 54 pictures representing the semantically matched counterparts from experiment 1 were included, resulting in a total of 108 pictures, half of which were presented during the encoding task.

We assessed the influence of the three experimental conditions on recognition performance. Therefore, the same model specified for the free recall was used (see Materials and Methods Experiment 2), but with the recognition performance as the dependent variable. One subject did not fully complete the recognition task, leading to 63 subjects being considered for analyses (31 females; mean age = 23.19, *SD* = 3.90, range 18–32).**Supplementary Results**

## Effects of fixation frequency and location on recognition performance, Experiment 2

Regarding passive recognition performance, there was a positive main effect of the factor ‘experimental condition’ (*F*(2,124)= 26.54, *p* = 2.5e-10). Post-hoc tests revealed an average decrease of recognized pictures in the ‘Area of no Interest‘ condition compared to the ‘Guided Fixation‘ condition by 16% (*t*(62)= -6.13, *p* = 1.4e-07. *R^2^β** = .074, 95% CI [.011, .180]). There was no evidence for a higher amount of recognized pictures in the ‘Guided Fixation × 2‘ condition compared to the ‘Guided Fixation‘ condition (*t*(62)= -.18, *p* = .85, *R^2^β** = .000, 95% CI [.000, .040]) (see Supplementary Fig. S2).

# Supplementary Discussion

In Experiment 1 the number of fixations in semantically informative picture areas were not only positively correlated to free recall, but also to recognition performance (see Supplementary Table S3). In experiment 2 we found that manipulating the scan path again affects both types of memories. However, a dissociation of the effects of fixation location and frequency was revealed. Both for free recall and recognition performance, it was beneficial to sample from semantically informative regions of the pictures during encoding. Only for free recall performance, however, it was beneficial if informative regions were sampled twice within a given time. A higher sampling frequency might promote associations between different regions of a picture (Wolfe and Horowitz 2017). We argue that free recall of episodic memories is likely to depend more on the association of visual areas of a picture than recognition, which can be achieved solely based on familiarity of isolated semantic areas of an image without requiring associations between them (Heisz et al. 2013). The finding of a positive correlation between fixation frequency and recognition memory in experiment 1 might be due to the high correlation of fixation frequency and number of semantic regions visited, with the latter being the actual driving factor.

Supplementary References

Boghen D, Troost BT, Daroff RB, Dell’Osso LF, Birkett JE. 1974. Velocity characteristics of normal human saccades. Invest Ophthalmol. 13(8):619-623.

Desikan RS, Ségonne F, Fischl B, Quinn BT, Dickerson BC, Blacker D, Buckner RL, Dale AM, Maguire RP, Hyman BT, et al. 2006. An automated labeling system for subdividing the human cerebral cortex on MRI scans into gyral based regions of interest. NeuroImage. 31(3):968-980. doi:10.1016/j.neuroimage.2006.01.021.

Dice LR. 1945. Measures of the Amount of Ecologic Association Between Species. Ecology. 26(3):297-302. doi:10.2307/1932409.

Fischl B, Salat DH, Busa E, Albert M, Dieterich M, Haselgrove C, van der Kouwe A, Killiany R, Kennedy D, Klaveness S, et al. 2002. Whole brain segmentation: automated labeling of neuroanatomical structures in the human brain. Neuron. 33(3):341-355.

Heisz JJ, Pottruff MM, Shore DI. 2013. Females Scan More Than Males: A Potential Mechanism for Sex Differences in Recognition Memory. Psychol Sci. 24(7):1157-1163. doi:10.1177/0956797612468281.

Luksys G, Fastenrath M, Coynel D, Freytag V, Gschwind L, Heck A, Jessen F, Maier W, Milnik A, Riedel-Heller SG, et al. 2015. Computational dissection of human episodic memory reveals mental process-specific genetic profiles. Proc Natl Acad Sci. 112(35):E4939-E4948. doi:10.1073/pnas.1500860112.

Mair P, Wilcox R. 2019. WRS2: A Collection of Robust Statistical Methods. https://CRAN.R-project.org/package=WRS2.

Santella A, DeCarlo D. 2004. Robust clustering of eye movement recordings for quantification of visual interest. ACM Press. p. 27-34.

Siegle GJ, Steinhauer SR, Stenger VA, Konecky R, Carter CS. 2003. Use of concurrent pupil dilation assessment to inform interpretation and analysis of fMRI data. NeuroImage. 20(1):114-124. doi:10.1016/S1053-8119(03)00298-2.

Wilcox RR. 2012. Modern statistics for the social and behavioral sciences: a practical introduction. Boca Raton: Taylor & Francis.

Wolfe JM, Horowitz TS. 2017. Five factors that guide attention in visual search. Nat Hum Behav. 1(3):0058. doi:10.1038/s41562-017-0058.

# Supplementary Tables

## Table S1.

Positive and negative modulation of activation by the number of fixations in AOIs during the encoding task in experiment 1.

|  | **Cluster**  **region names** | **Peak voxel**  **region name** | **Cluster** | |  | **Voxel** | | | | **EFA** | **ERA** |
| --- | --- | --- | --- | --- | --- | --- | --- | --- | --- | --- | --- |
|  |  |  | ***P_corr_*** | ***n*** |  | ***T_peak_*** | **MNI_peak_** | | | **%*n***  ***[k]*** | **%*n***  ***[k]*** |
|  |  |  |  |  |  |  | **X** | **Y** | **Z** |  |  |
| **Positive Modulation** | Precuneus (L)  Lingual gyrus (L)  Isthmus cingulum (L)  Precuneus (R) | Pericalcarine gyrus (L) | < .001 | 995 |  | 13.40 | -11 | -82.5 | 4 | 28.6  [285] | 19.7  [196] |
|  | Superior frontal cortex (L)  Rostral anterior cingulum (L)  Medial orbitofrontal cortex (L)  Superior frontal cortex (R)  Caudal anterior cingulum (R) | Medial orbitofrontal cortex (L) | < .001 | 643 |  | 8.09 | -2.75 | 55 | -4 | 8.6  [81] | 33.6  [216] |
|  | Inferior parietal cortex (L) | Inferior parietal cortex (L) | < .001 | 331 |  | 8.73 | -46.8 | -77 | 32 | 9.7  [11] | 0.0  0 |
|  | Parahippocampus (L)  Fusiform gyrus (L)  Lingual gyrus (L) | Parahippocampus (L) | < .001 | 97 |  | 8.56 | -22 | -38.5 | -12 | 28.9  [28] | 13.4  [13] |
|  | Superior frontal cortex (L)  Rostral middle frontal cortex (L) | Superior frontal cortex (L) | < .001 | 55 |  | 6.45 | -19.2 | 33 | 44 | 50.9  [28] | 21.8  [12] |
|  | Cerebellar cortex (R) | Cerebellar cortex (R) | .001 | 43 |  | 5.32 | 11 | -85.2 | -36 | 79.1  [34] | 9.3  [4] |
|  | Thalamus (L)  Thalamus(R) | Thalamus (L) | .006 | 25 |  | 5.78 | 0 | -5.5 | 8 | 4.0  [1] | 0.0  [0] |
|  | Parahippocampus (R) | Parahippocampus (R) | .046 | 13 |  | 5.75 | 22 | -33 | -16 | 7.7  [1] | 7.7  [1] |
|  |  |  |  |  |  |  |  |  |  |  |  |
| **Negative Modulation** | Cuneus (R)  Superior parietal cortex (R)  Lingual gyrus (R)  Cuneus (L)  Superior parietal cortex (L) | Cuneus (R) | < .001 | 964 |  | 15.6 | 5.5 | -82.5 | 28 | 0.0  [0] | 0.0  [0] |
|  | Superior parietal cortex (L)  Supramarginal gyrus (L)  Postcentral gyrus (L) | Superior parietal cortex (L) | < .001 | 524 |  | 8.30 | -22 | -49.5 | 68 | 0.0  [0] | 0.0  [0] |
|  | Lateral occipital cortex (L)  Inferior temporal cortex (L)  Middle temporal cortex (L) | Inferior temporal cortex (L) | < .001 | 123 |  | 6.51 | -49.5 | -66 | 0 | 0.0  [0] | 0.0  [0] |
|  | Precentral gyrus (L)  Superior frontal cortex (L)  Caudal middle frontal cortex (L) | Superior frontal cortex (L) | < .001 | 108 |  | 7.34 | -24.8 | -5.5 | 56 | 0.0  [0] | 0.0  [0] |
|  | Superior parietal cortex (R) | Superior parietal cortex (R) | < .001 | 88 |  | 6.66 | 24.8 | -49.5 | 64 | 0.0  [0] | 0.0  [0] |

Clusters with a voxel threshold of > 10 lying > 60% outside of cerebral white matter are reported. Cluster region name: Anatomical regions contributing a minimum of 5% to a given cluster are listed, ordered by the magnitude of the contribution. Regions are in accordance with the in-house atlas; Peak voxel region name: highest value in the cluster that is not lying in cerebral white matter is indicated; *P_corr_* represents the whole-brain *FDR*-corrected cluster *p* value, n the number of voxels in the cluster, *T_peak_* the *t* value of the peak voxel and [x,y,z] its coordinates in the MNI-space. EFA: Encoding-free-recall-association. Indicating in relative (%*n*) and absolute terms *[k]* how many voxels of a given cluster show a positive association between the encoding signal and free recall performance. ERA: Encoding-recognition-association. Indicating in relative (%*n*) and absolute terms *[k]* how many voxels of a given cluster show a positive association between the encoding signal and passive recognition performance.**Table S2.**

Intercorrelations of z-standardized eye tracking parameters in experiment 1.

|  | *N_fix_* | *N_fix_* in  AOIs | *N_AOIs_* | Blink duration | Interfixation distance |
| --- | --- | --- | --- | --- | --- |
| *N_fix_* | 1 |  |  |  |  |
| *N_fix_* in AOIs | .82 | 1 |  |  |  |
| *N_AOIs_* | .73 | .91 | 1 |  |  |
| Blink duration | -.22 | -.29 | -.30 | 1 |  |
| Interfixation distance | -.21 | -.34 | -.23 | .10 | 1 |

|  | **Eye tracking parameter** | **Main effect** |  |  | **Valence interaction** |  |  |  |  |  |
| --- | --- | --- | --- | --- | --- | --- | --- | --- | --- | --- |
|  |  | ***t***  **(*df*)** | ***p*** | ***R^2^β****  **95% CI []** | ***F***  **(*df*)** | ***p*** | **Posthoc-test** | ***t***  **(*df*)** | ***p*** | ***R^2^*** |
| **Passive recognition** | *N_fix_* | 4.50  (1339) | 2.9e-05 *** | .014  [.006, .026] | 3.16  (2,1339) | .06 |  |  |  |  |
|  | *N_fix_* in AOIs | 3.41  (1322) | .001 | .004  [.000, .011] | 5.67  (2,1322) | .014 * | negative | 3.38 (676) | .001 | .017 |
|  |  |  |  |  |  |  | neutral | 3.47 (675) | .001 | .018 |
|  |  |  |  |  |  |  | positive | 1.64  (658) | .10 | .004 |
|  | Blink duration | -0.57  (1291) | .57 | .000  [.000, .006] | 0.48  (2,1291) | .62 |  |  |  |  |
|  | Interfixation distance | -1.72  (1337) | .11 | .000  [.000, .005] | 4.73  (2, 1337) | .018 * | negative | -1.33 (679) | .27 | .003 |
|  |  |  |  |  |  |  | neutral | -0.12 (680) | .91 | .000 |
|  |  |  |  |  |  |  | positive | 1.59  (666) | .27 | .004 |

**Table S3.**

Models regressing eye tracking parameters on recognition performance in experiment 1.

# Supplementary Figures


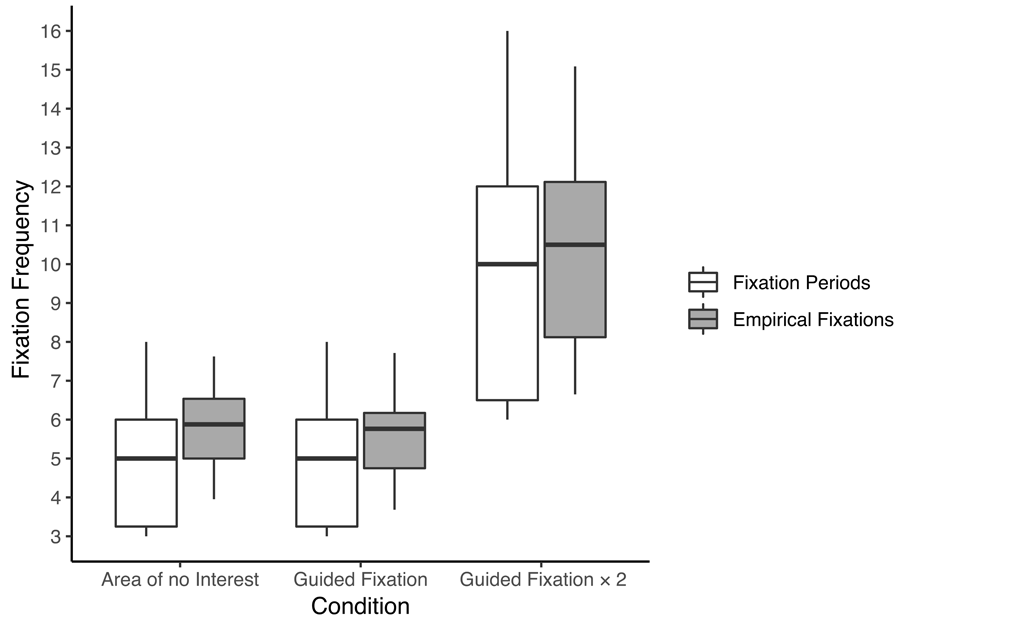


## Supplementary Fig. 1: Comparison between fixation periods and empirical fixations

We compared the fixation periods, given by the moving circle, and the empirically measured fixation frequency for each of the 54 pictures in 64 subjects. The figure shows the general level of empirical fixations to be slightly higher, suggesting that one fixation period usually is accompanied by more than one fixation. While the frequency of fixations was expected to be doubled in the ‘Guided Fixation × 2’ compared to the ‘Area of no Interest’ and the ‘Guided Fixation’ condition, the actual difference was slightly lower. The average increase in the ‘Guided Fixation × 2’ condition is 1.75 (*SD* = 0.18) compared to the ‘Area of no Interest’ condition and 1.82 (*SD* = 0.16) compared to the ‘Guided Fixation’ condition.


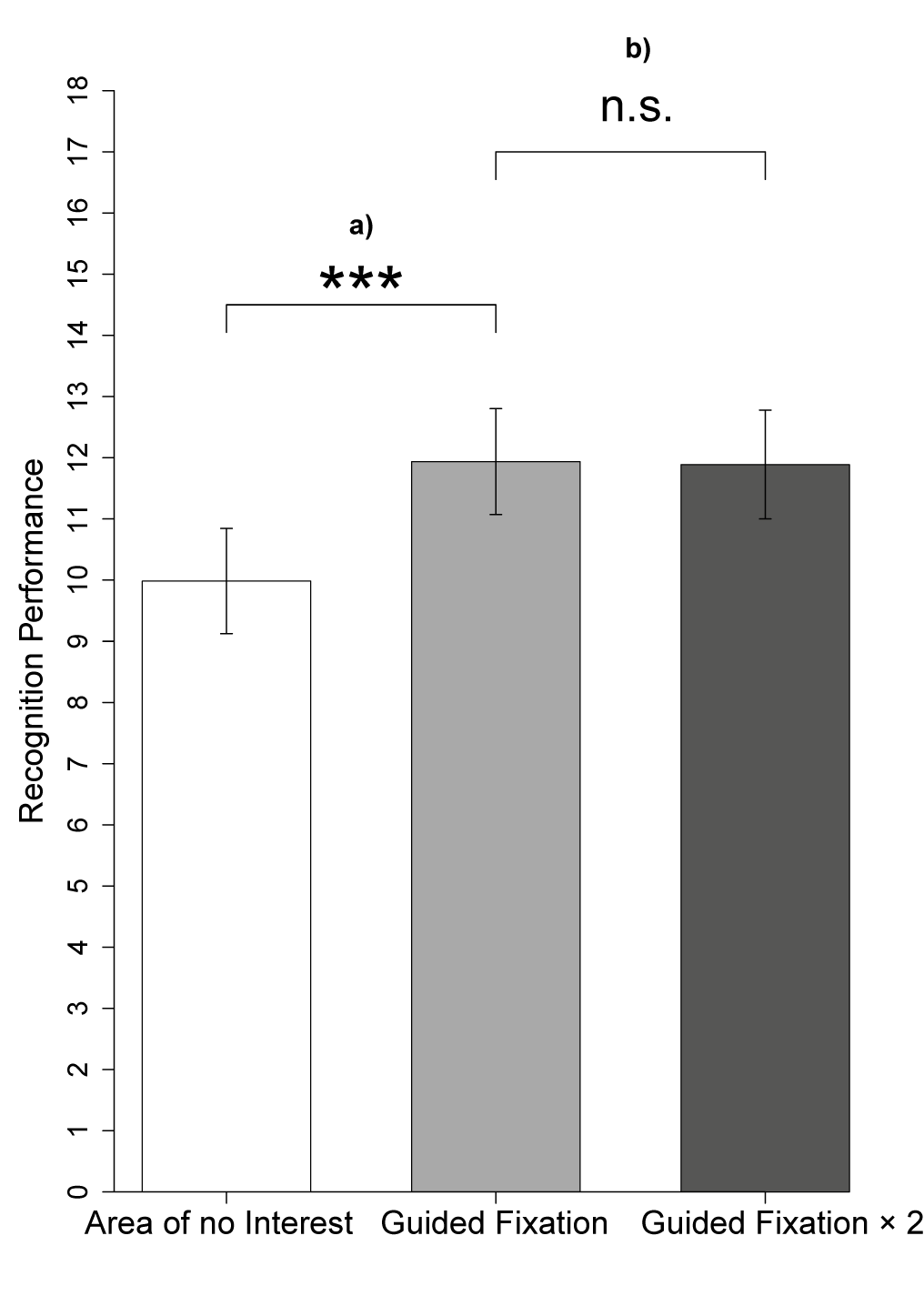


## Supplementary Fig. 2: Effect of scan path manipulation on recognition performance

Recognition memory effect in 63 subjects by a) decreasing the number of AOIs covered by fixations, leading to a lower amount of freely recalled pictures in the ‘Area of no Interest’ condition (*M* = 9.98, *SE* = 0.86) compared to the ‘Guided Fixation’ condition (*M* = 11.94, *SE* = 0.87) but not by b) increasing fixation frequency, leading to no significant differences in the ‘Guided Fixation × 2’ condition (*M* = 11.89, *SE* = 0.89) compared to the ‘Guided Fixation’ condition.
